# Unusual Case of Cleft Palate

**Published:** 1884-10

**Authors:** 


### UNUSUAL CASE OF CLEFT PALATE.

An interesting case is reported by A. Ernest Maylard, which was observed in the Infirmary of Glasgow. (*Lancet, Nov.* 24). The child, a male, aged four months, was found to have, besides a left hare-lip, a division in the alveolus immediately behind it. This extended directly backward and widened into quite an interval, but did not deviate toward the median line. On the opposite of the hard palate another parallel cleft existed, exactly in a corresponding situation. The structure between the two was about one-fourth of an inch wide, and was continued into the posterior wall of the pharynx. It was bony in structure. A bony septum seemed to extend upwards from it, as a probe could not pass in one cleft and out the other, bony resistance being met.

